# Exosome-Derived Mediators as Potential Biomarkers for Cardiovascular Diseases: A Network Approach

**DOI:** 10.3390/proteomes9010008

**Published:** 2021-02-01

**Authors:** Liliana Moreira-Costa, António S. Barros, André P. Lourenço, Adelino F. Leite-Moreira, Rita Nogueira-Ferreira, Visith Thongboonkerd, Rui Vitorino

**Affiliations:** 1 Department of Surgery and Physiology, Cardiovascular R&D Center, Faculty of Medicine of the University of Porto, Alameda Professor Hernâni Monteiro, 4200-319 Porto, Portugal; asbarros@med.up.pt (A.S.B.); aplourenco@yahoo.com (A.P.L.); a.f.leitemoreira@gmail.com (A.F.L.-M.); ritaferreira410@gmail.com (R.N.-F.); 2 Department of Cardiothoracic Surgery, Centro Hospitalar Universitário São João, Alameda Professor Hernâni Monteiro, 4200-319 Porto, Portugal; 3 Medical Proteomics Unit, Office for Research and Development, Faculty of Medicine Siriraj Hospital, Mahidol University, Bangkok 10700, Thailand; thongboonkerd@dr.com; 4 Department of Medical Sciences, Institute of Biomedicine (iBiMED), University of Aveiro, Campus Universitário de Santiago, Agra do Crasto, 3810-193 Aveiro, Portugal

**Keywords:** biomarkers, cardiovascular diseases, coronary artery disease, exosomes, heart failure

## Abstract

Cardiovascular diseases (CVDs) are widely recognized as the leading cause of mortality worldwide. Despite the advances in clinical management over the past decades, the underlying pathological mechanisms remain largely unknown. Exosomes have drawn the attention of researchers for their relevance in intercellular communication under both physiological and pathological conditions. These vesicles are suggested as complementary prospective biomarkers of CVDs; however, the role of exosomes in CVDs is still not fully elucidated. Here, we performed a literature search on exosomal biogenesis, characteristics, and functions, as well as the different available exosomal isolation techniques. Moreover, aiming to give new insights into the interaction between exosomes and CVDs, network analysis on the role of exosome-derived mediators in coronary artery disease (CAD) and heart failure (HF) was also performed to incorporate the different sources of information. The upregulated exosomal miRNAs miR-133a, miR-208a, miR-1, miR-499-5p, and miR-30a were described for the early diagnosis of acute myocardial infarction, while the exosome-derived miR-192, miR-194, miR-146a, and miR-92b-5p were considered as potential biomarkers for HF development. In CAD patients, upregulated exosomal proteins, including fibrinogen beta/gamma chain, inter-alpha-trypsin inhibitor heavy chain, and alpha-1 antichymotrypsin, were assessed as putative protein biomarkers. From downregulated proteins in CAD patients, albumin, clusterin, and vitamin D-binding protein were considered relevant to assess prognosis. The Vesiclepedia database included miR-133a of exosomal origin upregulated in patients with CAD and the exosomal miR-192, miR-194, and miR-146a upregulated in patients with HF. Additionally, Vesiclepedia included 5 upregulated and 13 downregulated exosomal proteins in patients in CAD. The non-included miRNAs and proteins have not yet been identified in exosomes and can be proposed for further research. This report highlights the need for further studies focusing on the identification and validation of miRNAs and proteins of exosomal origin as biomarkers of CAD and HF, which will enable, using exosomal biomarkers, the guiding of diagnosis/prognosis in CVDs.

## 1. Introduction

Cardiovascular diseases (CVDs) are the leading cause of morbidity and mortality worldwide [1]. Despite the advances in treatment approaches over the past decades that have considerably reduced the mortality, the underlying molecular mechanisms remain largely unknown. Moreover, the development of new diagnostic and therapeutic approaches is hampered by the complex nature of CVDs, including risk factors, such as obesity, diabetes mellitus, and hypertension [2]. The identification of novel biomarkers that can aid diagnosis and direct therapeutic strategies is a key target to address in order to achieve further reduction of morbidity and mortality rates [3].

Exosomes are small endosomal-derived membrane vesicles (30–150 nm diameter) [4] that have drawn the attention of researchers in the past decade for their relevance in intercellular communication [5]. In recent years, exosomes have been described in numerous cell–cell and cell–environment communications through the release of biological and/or chemical molecules to recipient cells. They govern physiological processes and show typical disturbances, which may constitute mechanistic pathways in various disease processes [6]. The biological content of exosomes, such as proteins and microRNAs (miRNAs), is conditioned by the cellular origin, function, and current state as a fingerprint of the donor cell. Moreover, exosomes provide some target site specificity to their cargos. Thus, exosomes better reflect cellular and regulatory processes than free circulating markers [7]. Moreover, most circulating miRNAs are enclosed in microvesicles, primarily exosomes, and exosome isolation improves the sensibility of miRNA amplification from biological fluids [8]. Evidence supports a potential role of exosomes as CVDs biomarkers, since profound changes in exosomal composition take place in CVDs, which are commonly attributed to shifts in cell type of origin and/or stimuli during biogenesis. Nevertheless, translation to clinically meaningful studies and clinical practice is lacking, mostly due to inconsistent exosomal isolation methods [9].

In this report, we summarize current knowledge on exosomal biogenesis, features, and functions, as well as the different available exosomal isolation techniques. Moreover, the potential use of exosome-derived mediators for diagnostic/prognostic purposes in CVDs is also emphasized through integrative network analysis.

### 1.1. Biogenesis, Characteristics, and Functions of Exosomes

#### 1.1.1. Exosomal Biogenesis

Exosomal biogenesis occurs within the endosomal system. Following endocytosis, early endosomes develop to multivesicular bodies (MVBs), and the cargo sequestered to the limiting membrane is selectively incorporated into intraluminal vesicles (ILVs) upon inward budding of the MVBs membrane. Most ILVs are released into the extracellular space as exosomes upon MVBs fusion with the plasma membrane or these components are trafficked to lysosomes for degradation [10]. Although the molecular mechanisms underlying these processes are still not fully understood, cargo sorting and packaging into ILVs occurs in a regulated mode [11]. Two pathways are proposed: one that requires the endosomal sorting complex required for transport (ESCRT) machinery (or ESCRT dependent) and another that is ESCRT independent.

ESCRT that consists of four separate proteins (ESCRT-0, -I, -II, and -III) works with vacuolar protein sorting-associated protein 4 (Vps4) to recognize ubiquitinated proteins and induce the invagination of the MVBs membrane, leading to the generation of ILVs [12]. ESCRT also seems to regulate exosomal release. As described by Ailawadi et al. [5], different ESCRT components and ubiquitinated proteins have been identified in isolated exosomes from various cell types. The depletion of ESCRT-0 hepatocyte growth factor-regulated tyrosine kinase substrate (Hrs) reduced the secretion of exosomes in HeLa cells [13], in dendritic cells [14], and also in HEK293 cells [15]. Conversely, ESCRT-III or the associated programmed cell death 6-interacting (ALIX) protein silencing increased exosomal secretion in HeLa cells [13], indicating that ALIX affects the nature of secreted vesicles. Moreover, ALIX is also required for the sequestration of transmembrane protein syndecans and, through its interaction with syntenies, participates in exosomal cargo selection [16]. However, the mechanisms involved in exosomal biogenesis and exosomal cargo sorting might change depending on the cell type and content [13].

As for the recently described ESCRT-independent pathway, it seems to depend on the lipid components of endosomal membranes, which include lipid rafts thought to be highly enriched in sphingomyelinases, afterward converted to ceramide by neutral sphingomyelinase 2 (nSMase2). The cone-shaped structure of ceramide and its accumulation causes spontaneous curvature of the endosomal membrane and coalescence of microdomains, which triggers the formation of ILVs [17]. Phospholipase D (PLD) is present in endosomal compartments and exosomes, and its activity regulates exosomal secretion [18]. PLD2 is responsible for the biosynthesis of lysophosphatidic acid (LPA), which, in turn, promotes inward budding of vesicles and ILVs formation by interacting with ALIX [19]. In addition to lipids, proteins are also essential players in ESCRT-independent exosomal biogenesis. Tetraspanins are the transmembrane proteins highly present in exosomes that, through interaction with other transmembrane proteins, cytosolic proteins, and lipids, organize membranes into tetraspanin-enriched microdomains (TEMs). For instance, tetraspanin CD9 and CD82 were reported to enhance the exosomal release of β-catenin from HEK293 cells [20], while CD63 is thought to be responsible for the loading of Epstein–Barr virus (EBV) encoded latent membrane protein 1 (LMP1) into exosomes [21]. Moreover, another protein that has been suggested to play a role in exosomal formation is the small integral membrane protein of the lysosome/late endosome (SIMPLE). This protein is not only present in the ILVs and exosomes, but its overexpression is also known to increase exosomal biogenesis, and exosomal accumulation of ALIX and CD63 [22]. ESCRT-dependent and ESCRT-independent pathways may also work synergistically, ensuring the specific sorting of bioactive molecules into exosomes [23].

#### 1.1.2. Sorting of Cargo into Exosomes

Exosomes deliver a complex cargo of proteins, lipids, and nucleic acids to target cells. Their compositions are, to some degree, conditioned by the cell type of origin and current state, suggesting the potential of exosomes to become useful biomarkers for disease diagnosis, prognosis, and treatment [7]. Due to their endosomal origin, exosomes are highly enriched in proteins that participate in exosomal biogenesis, releasing tetraspanins and ALIX, along with proteins responsible for membrane transport and fusion, such as annexins [24]. How cytosolic components are recruited into exosomes is still unclear; however, it is thought to involve the association of exosomal membrane proteins with chaperones (e.g., HSP70 and HSP90), engaged in antigen binding and presentation and found in exosomes derived from most of the cell types [25]. Therefore, certain specifically enriched members are widely used as exosomal marker proteins, namely TSG101, HSP70, CD81, and CD63 [24].

A major finding showed that exosomes also contain different patterns of RNAs, including both mRNA and miRNAs, and that exosomes-derived mRNAs could be translated into proteins by the target cells. The miRNAs packed into exosomes regulate gene expression and can undergo unidirectional transfer between cells, resulting in an effective and efficient intercellular communication [5]. This allows cells to induce transient or persistent phenotypic changes of recipient cells [26], revealing possible pathological mechanisms for disease progression, or potential underlying molecular mechanisms that can be studied for new treatment strategies in several diseases. According to Li et al. [27], miRNAs are the most abundant human plasma-derived exosomal RNA species. For instance, miRNAs, such as miR-214, miR-29a, miR-1, miR-126, and miR-320, are thought to participate in angiogenesis, hematopoiesis, exocytosis, and tumorigenesis, and have been reported in exosome-based cell-to-cell communications [7]. Even though several studies were done on cancer research using RNA sequencing analysis in different cancer cell lines [27], there is an emerging need of exosomal research for CVDs to gauge how exosomes modulate cell response to cellular stress during pathological challenges, such as hypoxia or extreme loading states.

#### 1.1.3. Characterization of Exosomes

The general characterization of exosomes includes the identification, description, and analysis of marker proteins, typically based on morphology, size, and flotation density. It is often challenging due to the method applied for exosomal isolation from conditioned cell culture media or body fluid samples. For instance, if heterogeneity is present in exosomal isolate, it will result in a mixed size distribution and also difficulties in profiling the cargo contents [7].

The description of the exosome-enriched proteins, namely transmembrane proteins and cytosolic proteins with membrane binding capacity (such as TSG101, HSP70, CD81, and CD63), can be done using Western blotting, flow cytometry, or global proteomic analysis using mass spectrometric techniques. Besides evaluating the levels of these proteins, which are already known to be present in exosomes, the presence of proteins not likely to be enriched in exosomes should also be determined [28].

Electron microscopy (EM) techniques are generally required to characterize exosomal morphology because particles are smaller than 300 nm [29]. Although transmission EM (TEM) is the standard tool, scanning EM (SEM) can be an alternative approach [30]. Both TEM and SEM studies highlight the heterogeneity of exosomal morphology and size [31,32], and both require ultracentrifugation to isolate exosomes but differ on the number of sample processing steps. Exosomes display a particular biconcave or cup-shaped morphology with a central depression on TEM while under SEM they are roughly spherical with a consistent size distribution [33]. It is thought that the characteristic central depression on TEM may be an artifact due to sample preparation owed to embedding in polymeric cellulose or membrane collapse during drying [25,33].

Although exosomes are commonly described as having diameters ranging from 40 to 100 nm, Wu et al. [33] characterized exosomes derived from mouse melanoma B16F0 cells that range from 139 to 158 nm. These discrepancies might be explained by the observation that exosomes appear to shrink over time [30], suggesting that the duration between exosomal isolation and characterization is relevant and should be standardly included in reports. In addition to EM, another common technique used for exosomal size determination is nanoparticle tracking analysis (NTA). This technique uses a video file, obtained from any microscopic technique capable of observing the movement and determining both the size and concentration of exosomes [34]. NTA has the advantage of being a fast and simple technique for the analysis of a large number of particles simultaneously. Therefore, it can be done at a relatively lower price when compared to TEM or SEM [35]. However, it cannot measure size or concentration when the exosomal number is below the NTA detection limit (approximately 1 × 10^7^ particles/mL) and does not differentiate an exosome from a protein aggregate of a similar size [36]. Moreover, recent advances in fluorescent microscopy (FM) provide non-invasive and direct approaches to image exosomes both in vitro and ex vitro. By tracking fluorescently labeled exosomal biomarkers, the dynamics of exosomal biogenesis, release, and uptake by the target cells can be revealed and studied in detail. Furthermore, FM allows more than two fluorescent dyes to stain and label different compartments of the cells simultaneously. This feature is particularly useful in the detection of the exosomal population. In general, exosomes can be visualized either by direct labeling of certain membrane proteins using fluorescent dyes or through fluorescent fusion proteins that are introduced in the host cell cytoplasm via transfection. Labeling with fluorescent dyes is convenient and can be adjusted easily; however, direct labeling often produces false-positive results by the excess of free dye [37].

One of the most defining characteristics of exosomes is their ability to float in density gradients. As reviewed by Théry et al. [38], exosomes typically have a density range from 1.13 to 1.19 g/mL in sucrose gradients. This wide density range reflects the heterogeneity of exosomes obtained by ultracentrifugation and supports a differential analysis of each fraction that can be accomplished by graded buoyant density centrifugation in which samples are centrifuged in sucrose gradient for different periods of time [39].

#### 1.1.4. Exosomal Functions

Depending on the cell type or tissue of origin, many distinct functions are recognized from exosomes. First reports focused on the ability of exosomes to eliminate plasma membrane proteins during maturation of reticulocytes into erythrocytes [40]. This pathway is beneficial to cells that do not have efficient degradation capability and are particularly efficient removing proteins that are resistant to conventional degradation by lysosomal proteases [41]. In physiological conditions, exosomes are also involved in facilitating immune response, antigen presentation, angiogenesis, and coagulation, among others [11].

One of the most relevant functions of exosomes is their ability to deliver macromolecular messages for cell-to-cell communications and signaling cascades. Cell-to-cell communications can be classified as contact dependent (via gap junctions and cell surface protein–protein interactions) or contact independent (via paracrine, endocrine, exocrine, or synaptic mechanisms) [24]. Exosomes mediate intercellular communication by direct ligand-receptor interaction, leading to activation of downstream signaling pathways, by extracellular cleavage of exosomal membrane proteins, by release of soluble ligands that bind to receptors of recipient cells, by direct membrane fusion with recipient cells with exosomal cargo release, and/or by internalization through endocytic mechanisms communication [5]. An advantage of exosome-mediated intercellular communication is the possibility to target multiple locations with particular specifications. Interestingly, exosomes do not randomly interact with any recipient cell, as because of their high expression levels of particular adhesion molecules (such as integrins and tetraspanins), their targets are well defined [42]. Selective transmission of exosomal cargo is a unique feature that renders exosomes valuable candidates for biomarker research. Circulating exosomes and their cell- or condition-specific cargos reflect ongoing cellular and molecular processes related to disease progression [7].

In recent years, evidence has suggested a new key role of exosomes in CVDs. Despite not being considered as typical secretory cells, cardiomyocytes, endothelial cells, and fibroblasts have been demonstrated to release exosomes, at least in an inducible manner as demonstrated by in vitro experiments [11]. The contents of released exosomes partly depend on the secretion stimuli. For instance, during hypoxia, cardiac exosomes are enriched with both angiogenic and pro-survival factors [43], and after myocardial infarction (MI), exosomes can inhibit cardiomyocyte apoptosis, induce cardiac repair, and promote local angiogenesis [44]. Exosomes are also thought to play a role in cardiac protection and repair. Indeed, cardiac progenitor cells (CPCs) have emerged as one of the most promising stem cell types for cardiac regeneration and repair since they can replace the apoptotic or dead cardiomyocytes by contractile cells in the adult heart and are able to release exosomes [45] that have protective effects against ischemic injury through the inhibition of apoptosis by the exosomal miR-21 in myocardial cells [46].

### 1.2. Exosomal Isolation Techniques

Exosomes have been characterized in almost all body fluids, including blood, urine, saliva, and pericardial fluid. To adequately characterize body fluid or tissue exosome composition, exosomes must first be isolated while preserving their structure and compositions and then analyzed according to size, morphology, concentration, biochemical composition, and cellular origin [29].

Currently, clinical translation is hampered by the lack of gold standard techniques to rapidly isolate, purify, and quantify exosomes [9]. Several approaches are available for exosomal isolation, such as ultracentrifugation- and size-based techniques, precipitation methods, immunoaffinity techniques, and microfluidics-based methods (Table 1), and the resulting exosomes fraction gives different yields, purity, and size depending on the method. The relevance of the approach depends on the sample source and intended use of exosomes. Moreover, additional challenges include the reproducibility and consistency of the resulting exosome isolates and the adequate quality control and standardization across research groups [7].

#### 1.2.1. Ultracentrifugation-Based Isolation Techniques

Ultracentrifugation is the preferred method for exosomal isolation, since it is easy to perform, requires little technical expertise, and consists of moderately time-consuming protocols, with little or no sample pre-treatment. However, due to the heterogeneity of exosomes and significant size overlap with other cellular compartments, this method is prone to contamination with other sample components, including lipoproteins and protein aggregates [52]. For a better separation and further purification of exosomes, the ultracentrifugation is widely combined with a sucrose density gradient strategy. Despite being the most common method, sucrose density gradient ultracentrifugation has disadvantages, such as the need of multiple centrifugations, risk of vesicle rupture and lower exosome yield due to high-velocity ultracentrifugation, and the requirement of large sample volumes. The latter may restrict application to cell culture media, since exosomes purified from urine or serum had a lower purity [47]. Ultracentrifugation-based isolation techniques thus could turn out to be inadequate for clinical studies on biomarkers [48,49].

#### 1.2.2. Size-Based Isolation Techniques

Size-based exosomal isolation techniques include both ultrafiltration and size-exclusion chromatography. Ultrafiltration is used to separate suspended particles or polymers using membrane filters with predefined molecular weight or size exclusion limits. Membrane filters with low protein-binding properties are more suitable for the isolation of exosomes, since they reduce the attachment of exosomal proteins and facilitate their isolation and recovery [50,51]. This method is faster than ultracentrifugation and requires no special equipment. Nevertheless, the use of force may result in the deformation and rupture of large vesicles, which may compromise the results of downstream analysis [52]. In size-exclusion chromatography, exosomes are separated according to their size while passing through a column packed with a heterosporous gel. The accessibility to the pores of the gel is differential since larger molecules can enter fewer pores than the smaller molecules and are eluted earlier [50]. For exosomal isolation, this method allows high efficiency in removing contaminants with good reproducibility. This approach, however, is laborious and has the potential for sample contamination with lipoproteins [53].

#### 1.2.3. Precipitation Methods

Precipitation methods are based on the low solubility of exosomes in solutions containing the hydrophilic polymer polyethylene glycol (PEG) [52]. This solution is most commonly provided in commercial kits and, therefore, is relatively easy to use with minimal cost. When compared to other methods, precipitation methods are faster and do not require specialized equipment. However, this method may co-precipitate non-vesicular contaminants, such as lipoproteins [54,55].

#### 1.2.4. Immunoaffinity-Based Techniques

The presence of different proteins and membrane receptors on the exosomal surface enables isolation by highly specific immunoaffinity-based techniques [52]. Since these techniques have high specificity, they can be used to study disease markers of diagnostic relevance expressed on the surface of exosomes and to reveal their origin and specialized function. They can be easily coupled with immunoassays and/or molecular diagnostic assays, such as microplate-based enzyme-linked immunosorbent assay (ELISA). Moreover, these methods recover highly pure and intact exosomes from complex biofluids still with a lower sample volume, cost, and hands-on time when compared to ultracentrifugation [56]. Nevertheless, immunoaffinity captures only the subset of exosomes expressing the antibody-recognized proteins on their surface, lowering the overall yield of the isolation process. Besides, since it is a relatively new approach, the best exosomal tags are yet to be established [52].

### 1.3. Exosomes in CVDs

During the past decade, the interest in the role of exosomes in both physiological and pathological conditions significantly increased. There is growing recognition of their function in CVDs pathogenesis through multifarious intercellular communication mechanisms that, nonetheless, have not yet been fully unraveled [11]. CVDs are the largest contributor to mortality worldwide, accounting for an estimated 17.9 million deaths and 31% of all global deaths in 2016. According to the World Health Organization (WHO), this number is expected to increase up to 23 million by 2030 [1]. Despite significant advances in medical management over the past decades, the five-year survival rate for CVDs is no longer improving [58]. Moreover, given their complex nature, including multiple risk factors, such as obesity, hypertension, diabetes, among others, the intricate molecular mechanisms underlying CVDs are far from being fully clarified. The identification of novel biomarkers remains vital to improve diagnosis and prognostic staging, as well as to guide therapy of CVDs with the goal of further reducing morbidity and mortality rates [59].

The human heart is mostly formed by cardiomyocytes; though, non-myocyte cell types, such as cardiac fibroblasts and endothelial cells, also have relevant roles in cardiac homeostasis [60]. The presence of diverse cell types within human cardiac tissue results in a complex intercellular network of numerous coordinated signaling pathways. These include cell–cell contacts, cell-extracellular–matrix interactions, and paracrine, autocrine, and endocrine effects of extracellular biologic and/or chemical molecules [6]. Exosomes contribute to these intercellular communications, although little is known about its regulation within the healthy and diseased hearts [9]. In recent years, accumulating evidence has implicated exosomes both in normal physiology (cardiac development, reticulocyte maturation, and myocardial angiogenesis) and in pathophysiological processes, including atherosclerosis, ischemia/reperfusion (IR) injury, and cardiac remodeling. For instance, stress conditions, such as hypoxia and inflammation, can modulate exosomal biological content and target cells, contributing to the improvement or impairment of heart function [43,61,62]. The biological contents and quantity of released exosomes change in pathological states, reflecting their cellular origin and the pathological disturbance. Since exosomes can be readily isolated from body fluids, allowing the characterization of both exosomes and their mediators, these might serve as non-invasive biomarkers for diagnosis and prognosis of CVDs [63,64]. To date, the potential role of exosomes has not been appraised in cardiovascular clinical research; therefore, further clinical research studies should be conducted to analyze the diagnostic and prognostic value as well as the functional role of exosomes in CVDs [3].

## 2. Material and Methods

### 2.1. Literature Search

**Figure 1:**
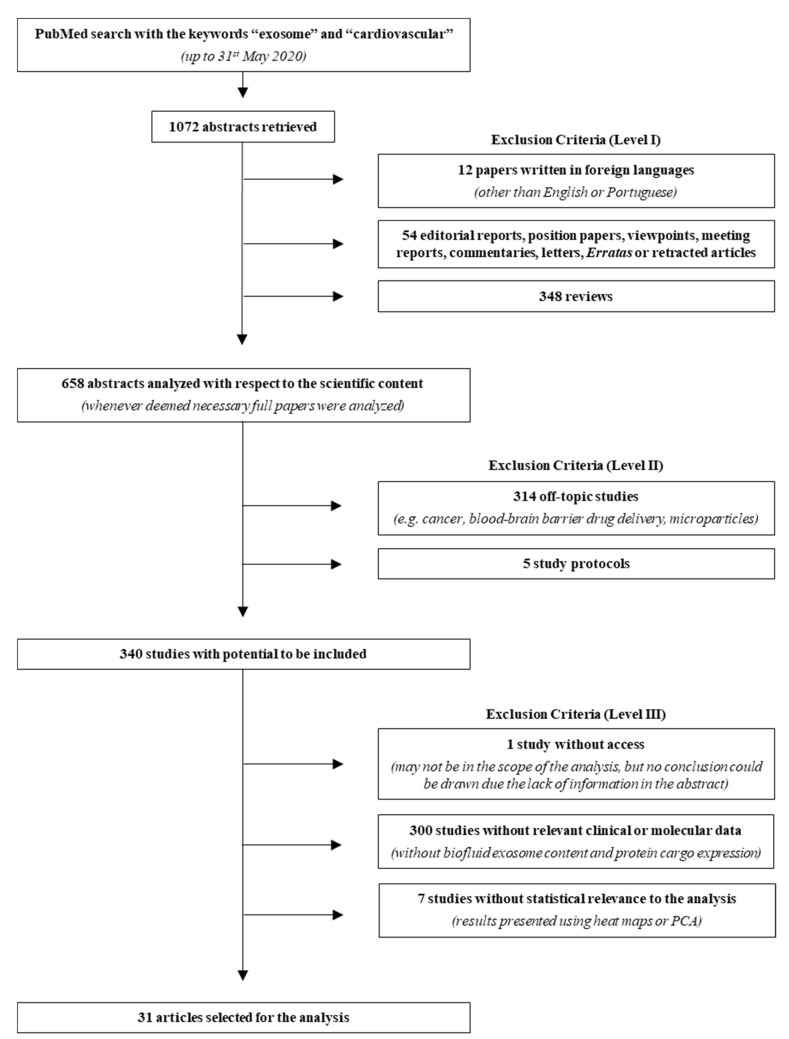
Search strategy flowchart. From the 1072 abstracts collected in PubMed, using the keywords “exosome” and “cardiovascular”, 31 reports were used for the meta-analysis and 1041 were excluded, according to the three criteria levels depicted in the right. Even though many studies described molecular analysis, namely using cell culture, these were not considered due to the lack of biofluid (plasma, serum, urine, pericardial fluid), exosome content description, protein cargo expression, and/or statistical relevance to the analysis. PCA, principal component analysis.

Aiming to explore all basic and clinical research studies on the interactions between exosomes and CVDs, a literature research was performed up to 31 May 2020 on PubMed using the keywords “exosome” and “cardiovascular”. A total of 1072 abstracts were retrieved and analyzed based on three levels of exclusion criteria (Figure 1, Table S1). For the first level, abstracts in other languages than English or Portuguese, editorial reports, position papers, viewpoints, meeting reports, commentaries, letters, erratas or retracted articles, and reviews were excluded. For the second level, off-topic abstracts and study protocols were excluded. Finally, the third level excluded studies in which access to relevant clinical or molecular data was not complete or studies in which statistical analysis was deemed inadequate. After analyzing the abstracts, 31 papers were validated for further evaluation. In order to extract proteins and miRNAs from exosomal origin and relevant to CVDs development, data from these articles were manually organized and each mediator was compared between healthy/non-diseased and diseased conditions.

### 2.2. Bioinformatic Analysis

In order to evaluate screened molecular species as potential biomarkers for CVDs, we conducted a network analysis considering the information gathered in Table 2. We studied the exosome-derived mediators present in plasma and serum of patients with (1) CAD, which included CAD, AMI, coronary artery dilatation due to KD, myocardial IR, and CABG; and (2) HF, including acute HF. Only studies reporting significant differences (*p* < 0.05) were considered. According to the criteria described above, all included studies characterized exosomal molecules that mediated CAD and HF, including miRNAs and proteins. We used Cytoscape v. 3.5.1 [65] to carry out a network analysis and FunRich functional enrichment analysis tool to confirm exosomal origin in Vesiclepedia (FunRich v. 3.1.3) [66,67]. Moreover, we used Cytoscape plugin ClueGo + CluePedia [68,69] tool in order to perform a functional analysis of the circulating exosomal proteins found to be up- or downregulated in CAD patients.

## 3. Results

### 3.1. Literature Search

Resultant of the performed literature search, 31 papers were manually curated, and each exosome-derived mediator was assessed in healthy/non-diseased and diseased conditions (Table S2). Table 2 summarizes the studies that were performed to evaluate the levels of miRNAs and proteins in different body fluids (plasma, serum, urine, and pericardial fluid) both in human and animal studies of (1) CVDs: coronary artery disease (CAD), acute MI (AMI), coronary artery dilatation due to Kawasaki disease, acute heart failure (HF) due to dilated cardiomyopathy, HF, and idiopathic pulmonary arterial hypertension; (2) CVDs risk factors (arterial disease, hypertension, obesity, type 2 diabetes, diabetic nephropathy, and obstructive sleep apnea) and (3) myocardial IR or coronary artery bypass graft (CABG).

As depicted from Table 2, most of the studies were performed in human samples, mainly plasma. The methodological approaches usually used for exosomal isolation were commercial kits for exosomal precipitation, such as ExoQuick Exosome Precipitation Solution commercial kit (System Biosciences, LLC; California, USA) and ultracentrifugation. For validation and characterization, most of these studies used Western blotting to analyze protein expression and EM techniques to examine exosomal morphology.

This literature search resulted in a relatively small number of clinical studies and larger pool of studies focused on the experimental development of therapeutic options for CVDs using exosomes (using cell culture and manipulation of animal models of CVDs). The lack of clinical translation hampers our understanding of the biologic functions and underlying molecular mechanisms of exosomes in CVDs. The paucity of clinical studies also highlights that the role of exosomes in CVDs is far from elucidated and warrants further studies to analyze the diagnostic and prognostic value and functional roles of exosomes in CVDs [3].

### 3.2. Circulating Exosomal miRNAs in Coronary Artery Disease and Heart Failure Patients

**Figure 2:**
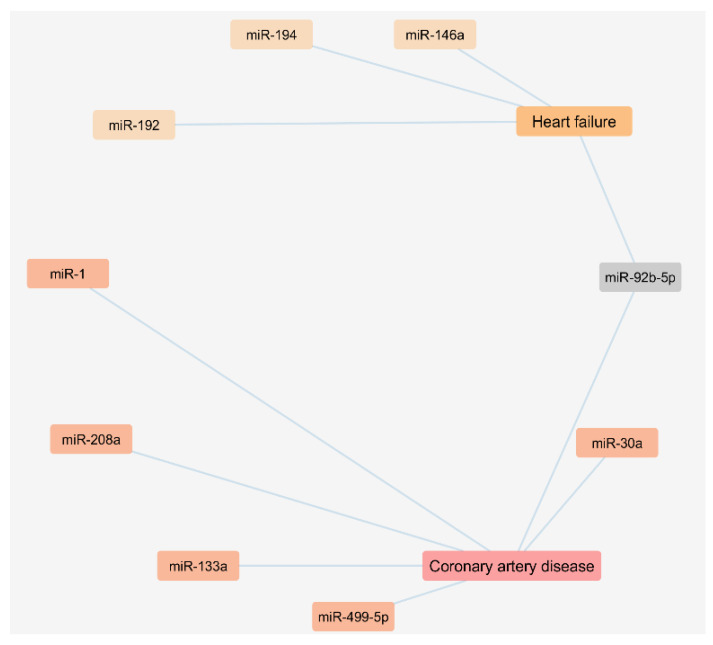
Cytoscape network of circulating miRNAs of exosomal origin found upregulated in patients with coronary artery disease and heart failure.

The performed literature search also enabled us to analyze a network regarding the exosomal miRNAs found to be up regulated in plasma or serum samples of CAD and HF patients as presented in Figure 2.

**Figure 3:**
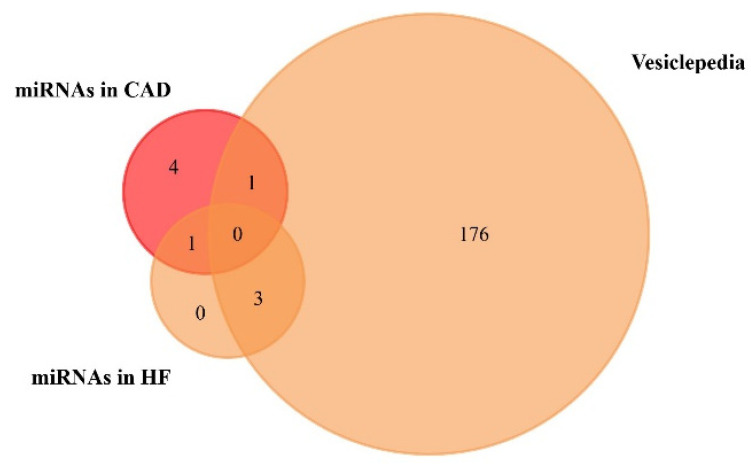
Venn diagram of the miRNAs found to be upregulated in coronary artery disease (CAD) and heart failure (HF), from plasma and serum human samples, using FunRich tool (accessed on 6 July 2020). From 6 upregulated miRNAs in CAD, 1 was included in the Vesiclepedia database. From 4 upregulated miRNAs in HF, 3 were included in the Vesiclepedia database.

We used the FunRich functional enrichment analysis tool to confirm exosomal origin in Vesiclepedia (FunRich v. 3.1.3). Vesiclepedia is a database of catalogued proteins, RNAs, lipids, and metabolites that have been identified in all classes of extracellular vesicles, including exosomes [68,69]. We found that one upregulated miRNA in CAD and three upregulated miRNAs in HF patients were included in the Vesiclepedia database (Figure 3, Table 3, Table S4 and Table S5). Moreover, a list of the top 10 target genes of exosome-based miRNAs is available on Table S3 of Supplementary Materials.

### 3.3. Circulating Exosomal Proteins in Coronary Artery Disease Patients

**Figure 4:**
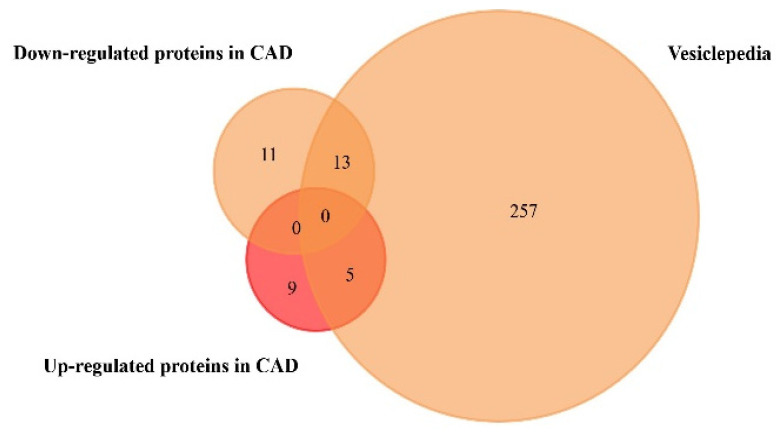
Venn diagram of the proteins found to be up- and downregulated in coronary artery disease, from plasma and serum human samples, using FunRich tool (accessed on 7 July 2020). From 14 upregulated and 24 downregulated proteins, 5 upregulated and 13 downregulated proteins were identified in the Vesiclepedia database.

We performed a similar analysis regarding the exosome-derived proteins found to be significantly up- or downregulated only in CAD, since the studies collected in Table 2 have no information regarding protein expression in plasma or serum samples of HF patients. Results are shown in Figure 4 and Table 4 (Supplementary Material on Tables S6 and S7). 

**Figure 5:**
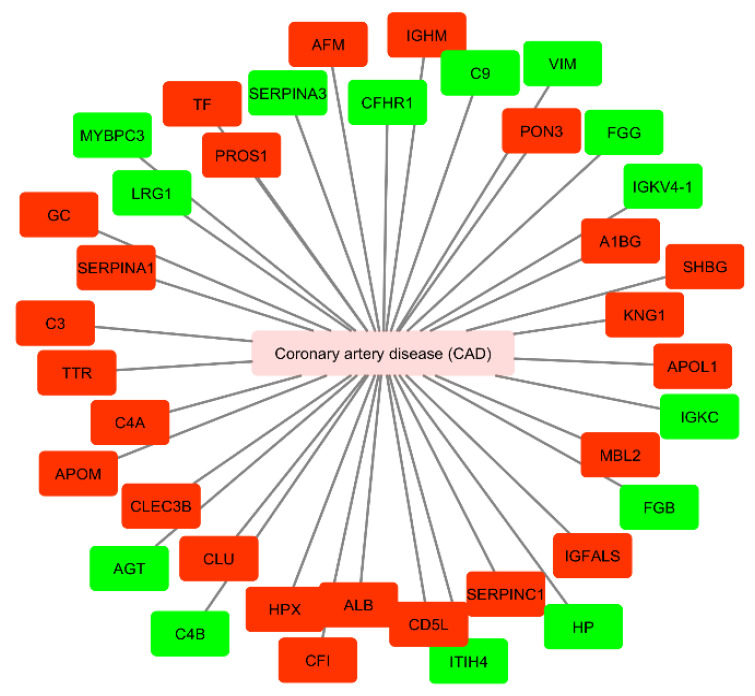
Cytoscape network of circulating proteins of exosomal origin found to be up- or down-regulated in patients with coronary artery disease. Mediators up-regulated are presented in green and down-regulated in red (accessed at 11th July 2020). CAD, coronary artery disease; AGT, angiotensinogen; C4B, complement C4-B; HP, haptoglobin; FGG, fibrinogen gamma chain; FGB, fibrinogen beta chain; VIM, vimentin; ITIH4, inter-alpha-trypsin inhibitor heavy chain H4; C9, complement component C9; IGKC, immunoglobulin kappa constant; MYBPC3, myosin-binding protein C, cardiac-type; SERPINA3, alpha-1 antichymotrypsin; LRG1, leucine-rich alpha-2-glycoprotein; CFHR1, complement factor H-related protein 1; IGKVA-1, immunoglobulin kappa variable 4-1; PROS1, vitamin K-dependent protein S; C4A, complement C4-A; A1BG, alpha-1B-glycoprotein; KNG1, kininogen-1; CLU, clusterin; C3, complement C3; CD5L, CD5 antigen-like; APOL1, apolipoprotein L1; TF, serotransferrin; ALB, serum albumin; MBL2, mannose-binding protein C; TTR, transthyretin; SERPINA1, alpha-1-antitrypsin; AFM, afamin; IGFALS, insulin-like growth factor-binding protein complex acid labile subunit; HPX, hemopexin; SERPINC1, antithrombin-III; GC, gene name of vitamin D-binding protein (DBP); CFI, complement factor I; APOM, apoliprotein M; CLEC3B, tetranectin; PON3, serum paraoxonase/lactonase 3; SHBG, sex hormone-binding globulin.

Moreover, we performed a network analysis to evaluate the association between circulating mediators from exosomal origin found to be up- or downregulated in CAD (Figure 5). We used UniProt identifiers for each protein.

**Figure 6:**
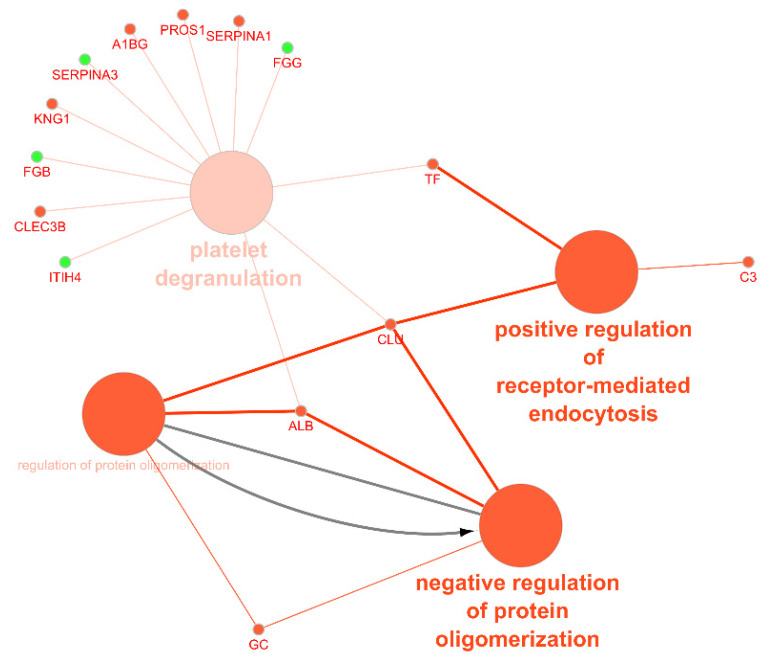
Functional analysis of the circulating exosomal proteins found to be up- or downregulated in coronary artery disease. Nodes in green represent upregulated proteins and nodes in red represent downregulated proteins. Arrow depicts direction of association (accessed at 12 July 2020). ITIH4, inter-alpha-trypsin inhibitor heavy chain H4; FGB, fibrinogen beta chain; SERPINA3, alpha-1 antichymotrypsin; FGG, fibrinogen gamma chain; CLEC3B, tetranectin; KNG1, kininogen-1; A1BG, alpha-1B-glycoprotein; PROS1, vitamin K-dependent protein S; SERPINA1, alpha-1-antitrypsin; TF, serotransferrin; C3, complement C3; CLU, clusterin; ALB, serum albumin; GC, gene name of vitamin D-binding protein (DBP).

**Figure 7:**
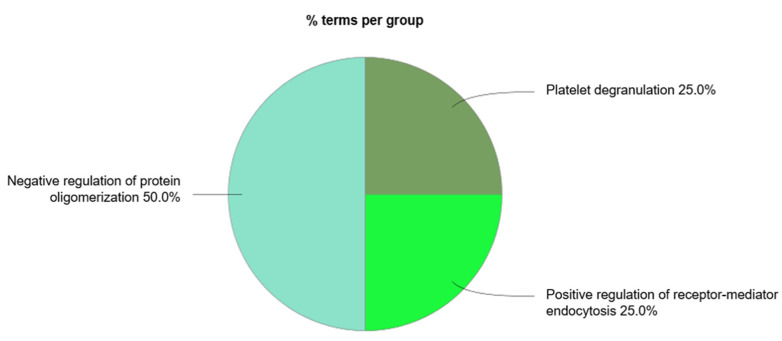
ClueGo + CluePedia analysis of the biological processes considering the exosomal proteins whose circulating levels were found to be downregulated in coronary artery disease (accessed at 12 July 2020).

We also performed a functional analysis of the circulating exosomal proteins found to be up- or downregulated in CAD patients. Figure 6 represents the most common functional processes associated to these mediators. In Figure 7, there is a representation of the main biological processes associated to the circulating exosomal proteins found to be downregulated in CAD.

There were four main biological processes associated to the circulating exosomal proteins studied: the positive regulation of receptor-mediated endocytosis, the regulation (and negative regulation) of protein oligomerization, and platelet degranulation. The protein of the complement signaling pathways complement C3 (C3), serotransferrin (TF), and clusterin (CLU) were found to participate in the positive regulation of receptor-mediated endocytosis. Together with CLU, serum albumin (ALB) and vitamin D-binding protein (DBP) were associated to the negative regulation of protein oligomerization. Circulating exosomal downregulated proteins CLU, TF, ALB, tetranectin (CLEC3B), kininogen-1 (KNG1), alpha-1B-glycoprotein (A1BG), vitamin K-dependent protein S (PROS1), and alpha-1-antitrypsin (SERPINA1) were found to participate in the platelet degranulation molecular mechanism. In addition, also the upregulated exosomal proteins inter-alpha-trypsin inhibitor heavy chain H4 (ITIH4), fibrinogen beta chain (FGB), alpha-1 antichymotrypsin (SERPINA3), and fibrinogen gamma chain (FGG) were found to be associated to this process.

## 4. Discussion

Although there is expanding interest in the role of exosomes in CVDs, their use as biomarkers in clinical research is still limited by diverse difficulties in exosome isolation and characterization [9]. Most of the conducted studies regarding exosomes and its role in CVDs, risk factors, and complications were performed by collecting plasma and isolating the exosomes by precipitation or ultracentrifugation. Mainly, these studies used the Western blotting method to analyze the protein expression and EM techniques to characterize the morphology of the isolated exosomes. In this report, we attempt to link exosomes and exosome-derived mediators to CVDs in an association network approach that gathers literature findings coupled with bioinformatics analysis of the results. The integration of these exosomal markers by the network approach allowed a wide-ranging analysis of the potential biomarker of these mediators in the scope of CVDs.

CAD is the most common type of heart disease and it results mainly from the activation of inflammatory, oxidative stress, and endothelial dysfunction pathways and enriched-cholesterol plaques in the arteries. The exosomes released from stem cells, endothelial cells, cardiomyocytes, and platelets, among others, include potential valuable biological information for the development and progression of CAD [101]. Currently, miRNAs are the most studied elements contained in exosomes for their potential as biomarkers. Some miRNAs are reported to be tissue specific, such as miR-208a and miR-499-5p, which are highly present in the heart tissue, and miR-1a and miR-133a, which are expressed in heart and skeletal muscles [70]. These miRNAs can also be found in the circulation and are thought to play a cardioprotective role, namely by decreasing hypertrophy and fibrosis after AMI [101]. In this work, we analyzed six circulating exosome-derived miRNAs found to be upregulated in patients with CAD: miR-133a, miR-208a, miR-1, miR-499-5p, miR-92b-5p, and miR-30a. The performed search in Vesiclepedia database uncovers if the evaluated miRNAs have already been identified in exosomes. Since only miR-133a was included in Vesiclepedia database, we can conclude that the other miRNAs analyzed in this study were not yet identified in exosomes or manually curated in this database. Moreover, these non-included miRNAs of exosomal origin can be proposed for further research. Taking into consideration the previously mentioned challenges in exosome isolation and characterization, the main limitation associated to the Vesiclepedia database is some uncertainty associated to the exact origin of the identified mediators [102]. Nevertheless, the use of this database allowed us to get a better comprehension of the existing literature regarding exosome and exosome-derived mediators.

Circulating exosomal miR-133a increased levels can be considered among the biomarkers for early diagnosis of AMI. miR-133a levels in blood are a result of their release from cardiomyocytes after cardiac injury and cell death. Moreover, the capture of miR-133a by adjacent surviving cells in infarcted cardiac areas contributes to the inhibition of hypertrophy [103,104]. According to Cheng et al. [70], exosome-derived miR-133a, together with miR-208a, miR-1, and miR-499, are found to be increased in the plasma of AMI patients. Moreover, an available clinical study reported increased circulating miR-133a and miR-1 levels in patients with acute coronary syndrome [103] despite lacking confirmation of their exosomal origin. Increased plasma levels of miR-208a and miR-499 are associated to cardiac damage in acute HF, acute viral myocarditis, and AMI [105]. miR-208a is encoded by the α-myosin heavy-chain gene and, therefore, has a particular role in cardiac contractility. High levels of miR-208a are associated to arrythmias, fibrosis, and hypertrophy growth [106,107].

miR-30a is responsible for the regulation of cardiomyocyte autophagy after hypoxia. According to Yang et al. [75], serum levels of miR30a with exosomal origin were found to be increased in AMI patients. The exosomes enriched with miR-30a in a hypoxic condition were thought to be transferred between cardiomyocytes in order to maintain an autophagic response. Nevertheless, this was still not validated in in vivo cardiac ischemia models [60,75]. Moreover, exosome-derived miR-92b-5p is also considered a putative biomarker since it was determined to be increased in patients of acute HF due to dilated cardiomyopathy [79] and in patients with HF [80]. This miRNA is believed to contribute to atrial fibrillation, which often coexists with HF [108].

Many CADs progress to a state of chronic HF, determined by the complex molecular mechanisms of cardiac remodeling and vascular dysfunction. One of the major challenges in HF management is to identify a reliable approach to evaluate the prognosis of the disease [109]. Despite the use of functional parameters and resulting risk stratification scores, prognosis evaluation among HF patients would beneficiate by an extended use of exosomes as biomarkers [58]. We were able to identify four circulating exosome-derived miRNAs upregulated in HF: miR-192, miR-194, miR-146a, and miR-92b-5p. From these, only miR-92b-5p was not included in Vesiclepedia database. Evidence highlights the future clinical applications of miR-192, miR-194, and miR-34a as predictive indicators of HF [110]. As reported by Matsumoto et al. [81], circulating increased levels of exosomal miR-192 and miR-194 are highly related to the development of HF after AMI. Additionally, despite not being significantly enriched, exosome fraction of HF patients shows a tendency of an increased level of miR-34a. Exosome-derived miR-92b-5p was found to be increased in patients with acute HF caused by dilated cardiomyopathy [79] and in patients with HF with reduced ejection fraction hospitalized for acute HF [80].

Besides the analysis regarding the circulating levels of exosome-derived miRNAs, we also analyzed the proteins of exosomal origin found to be up- or downregulated in plasma or serum samples of patients with CAD. We analyzed 14 upregulated proteins: angiotensinogen (AGT), complement C4-B (C4B), haptoglobin (HP), FGG, FGB, vimentin (VIM), inter-alpha-trypsin inhibitor heavy chain H4 (ITIH4), complement component C9 (C9), immunoglobulin kappa constant (IGKC), myosin-binding protein C, cardiac-type (MYBPC3), alpha-1 antichymotrypsin (SERPINA3), leucine-rich alpha-2-glycoprotein (LRG1), complement factor H-related protein 1 (CFHR1), and immunoglobulin kappa variable 4-1 (IGKVA-1). From these, only five proteins were identified in the Vesiclepedia database: AGT, C4B, HP, FGG, and FGB. Moreover, FGB, FGG, ITIH4, and SERPINA3 were found to be involved in the platelet degranulation molecular mechanism.

One of the main pathophysiological mechanisms associated to CAD is endothelial dysfunction. It is frequently observed in patients with cardiovascular risk factors and contributes to the development of atherosclerosis and myocardial ischemia. Endothelial dysfunction is characterized by impaired fibrinolysis, prothrombotic, and proinflammatory responses, which leads to the activation of coagulation factors and platelets [111]. According to Zhang et al. [78], the exosomal proteins FGB, FGG, ITIH4, and SERPINA3 were found to be upregulated in CAD. These proteins are associated to an alteration of the platelet degranulation and activation [112]. FGB is cleaved by thrombin to yield monomers that polymerize into insoluble fibrin, usually associated with coagulation disorders. Both exosomal FBG and FGG were found to be significantly increased in patients with malignant pulmonary nodules and evidence reveals its increasing interest of candidate diagnostic biomarkers for bladder and prostate cancer, among others [113]. ITIH4 is a protein involved in inflammatory responses and host–virus interaction processes. For instance, ITIH4 is elevated in urine samples of patients with type 2 diabetes mellitus and microalbuminuria and it is considered as a putative biomarker for diabetic kidney disease [114]. SERPINA 3 gene codes for alpha-1-antichymotrypsin, a powerful inhibitor of proteolytic enzymes. An increase in alpha-1-antichymotrypsin occurs during tissue damage and it is considered to be one of the “acute phase reactant” [115]. Moreover, it contributes to the impairment of the coagulation cascade and fibrinolysis in the development of calcific aortic stenosis and could be useful as a biomarker for this disease with considerable clinical value [116].

The downregulated circulating proteins CLU, TF, ALB, tetranectin (CLEC3B), kininogen-1 (KNG1), alpha-1B-glycoprotein (A1BG), vitamin K-dependent protein S (PROS1), and alpha-1-antitrypsin (SERPINA1) were found to participate in the platelet degranulation molecular mechanism. Exosomal CLU is a heterodimeric glycoprotein highly expressed after AMI in order to induce vascular growth and cardiac tissue regeneration [117]. ALB is the most abundant circulatory protein, associated with various physiological functions, such as the maintenance of microvascular integrity, regulating metabolic and vascular functions, and anticoagulant effects, among others [118]. Indeed, the association between low levels of ALB and increased risk of CVD [119] and HF [120] is reported in several studies, as well as the prognostic significance of this mediator in CAD [121]. Together with CLU and ALB, DBP was found to be associated to the negative regulation of protein oligomerization. DBP, also associated to the negative regulation of protein oligomerization, is found to be downregulated in plasma samples of patients with coronary artery atherosclerosis and can be used as a potential predictor factor of the severity of the disease [122].

The downregulated proteins C3, TF, and CLU participate in the positive regulation of receptor-mediated endocytosis, which suggests the association with exosomal biogenesis [10]. C3 is an important component of the complement system, involved in the immune response. When considering local and systemic inflammation, not only an increase in extracellular vesicles is observed but also an increase in complement activation products. This link between vesicles and the complement signaling pathway leads to innate and adaptive immune responses [123].

The present study suffered some limitations inherent to the applied methods. The limitations to the integrative analysis of data posed by a small number of heterogeneous studies, with different recruitment criteria, comorbidities, and etiologies of CVDs, raise important hurdles to this approach. Moreover, the diversity of miRNAs and proteins of exosomal origin found to be significantly expressed in CAD and HF patients also represents a limitation. Nevertheless, we aggregated the available studies on heterogeneous populations of HF patients (e.g., acute and chronic heart failure of diverse etiologies) in a common group. However, we were not able to refine our grouping strategy due to the low number of available studies for each of the potential subgroups.

## 5. Conclusions

The network analysis of both miRNAs and proteins of exosomal origin in CAD and HF allowed the identification of possible molecular biomarkers for the diagnosis of these diseases. The upregulated exosomal miRNAs miR-133a, miR-208a, miR-1, miR-499-5p, and miR-30a have been described mostly for the early diagnosis of AMI. The evaluation of HF development would benefit from using exosome-derived miR-192, miR-194, miR-146a, and miR-92b-5p as potential biomarkers. Vesiclepedia included one out of six upregulated miRNAs in patients with CAD and three out of four miRNAs upregulated in patients with HF. Despite not including studies with up- or downregulated exosomal proteins in HF patients, our study allowed the assessment of putative protein biomarkers in CAD patients. From the 14 upregulated exosomal proteins, FBG, FGG, ITIH4, and SERPINA3 coding gene for alpha-1-antichymotrypsin were associated to endothelial dysfunction and coagulation cascade activation and platelet degranulation. These are found to be relevant mainly in cancer clinical research and could be also largely studied in the scope of CVDs. Moreover, from 24 downregulated exosomal proteins, ALB, CLU, and DBP were found to be decreased in AMI, CAD, and HF patients and were associated to a prognostic value of these diseases. Five out of 14 upregulated proteins and 13 out of 24 downregulated proteins in patients with CAD were included in Vesiclepedia.

The integration of exosomes-based miRNAs and proteins, concerning the expression variation in several body fluids, allows a comprehensive analysis of the potential biomarker role of these components in CVDs. This report highlights the need for further studies that focus on the identification and validation of miRNAs and proteins of exosomal origin as biomarkers of CAD and HF. By doing this, the use of exosomal biomarkers may be considered as a new approach for diagnosis/prognosis of CVDs.

## Figures and Tables

**Table 1:** Main advantages and disadvantages of different exosomal isolation techniques.

Isolation Method	Main Advantages	Main Disadvantages	Ref.
Sucrose density gradient ultracentrifugation	Easy to perform, requires little technical expertise.	Time-consuming, risk of exosomal rupture and loss, requires a large volume of samples.	[47,48,49]
Size-based methods (i) Ultrafiltration	Fast method with no requirement for special equipment.	Risk of exosomal rupture and loss.	[50,51,52]
(ii) Size-exclusion chromatography	Preserves exosomal structure with high purity and good reproducibility.	Laborious, possible contamination with lipoproteins.	[50,53]
Precipitation	Easy to perform, minimal cost with no requirement for special equipment.	Risk of contamination with lipoproteins.	[54,55]
Immunoaffinity-based methods	Preserves exosomal structure with high purity, requires a small volume of samples with a low experiment time.	Low yield, exosomal tags need to be established.	[52,56]
Microfluidics-based methods	Preserves exosomal structure and compositions, requires a small volume of samples and reagent consumption at a low cost.	Lack of method validation, standardization and large-scale tests on clinical samples.	[49,52,57]

**Table 2:** Overview of published studies on body fluids-based exosomes and their mediators in CVDs, risk factors, and complications.

Cardiovascular Disease	Isolation Method	Biofluid	Discovery Cohort Size	Exosome Validation Method	Exosomes Characteristics	Biomarker Candidate(s)	Ref.
Coronary artery disease (CAD)	ExoQuick Exosome Precipitation Solution	Plasma	C57BL/6 mice, n = 5	Electron microscopy Nanoparticle tracking analysis Western Blotting	n. d.	miR-1, miR-208a, miR-133a, miR-499-5p	[70]
	Ultracentrifugation (sucrose)	Serum	Human, n = 5	Electron microscopy CD9 staining	n. d.	MYBPC3, VIM	[71]
	n. d.	Plasma	Tandem stenosis group, mice n = 4	n. d.	n. d.	miR-223, miR-339, miR-21	[72]
	Ultracentrifugation	Plasma	Tandem stenosis group, mice n = 4	Electron microscopy Western blotting	n. d.	miR-223, miR-339, miR-21	[73]
	Ultracentrifugation	Plasma	Human, n = 25	Mass spectrometry for proteome analysis, using nano–liquid chromatography LTQ Orbitrap XL mass spectrometer	n. d.	n. d.	[74]
Acute myocardial infarction (AMI)	ExoQuick Exosome Precipitation Solution	Plasma	C57BL/6 mice, n = 5	Electron microscopy Nanoparticle tracking analysis Western Blotting	n. d.	miR-1, miR-208a, miR-133a, miR-499-5p	[70]
	ExoQuick Exosome Precipitation Solution	Serum	Human, n = 28	Transmission electron microscopy Flow cytometry Immunoblotting	CD63 expression	miR-30a	[75]
	Ultracentrifugation	Plasma; Pericardial fluid	Human, n = 12	Western Blotting	Rab 5B and CD81 expression	n. d.	[76]
	Ultracentrifugation	Serum	Human, n = 10	n. d.	n. d.	Apo-J	[77]
Coronary artery dilatation due to Kawasaki disease (KD)	ExoQuick Exosome Precipitation Solution	Serum	Human, n = 6	Transmission electron microscopy Western blotting	CD9, CD81, and flotillin expression	ITIH4, PROS1, C9, AFM, A1BG IGFALS, C4A, HPX, SERPINC 1, Inter-alpha (Globulin) inhibitor H4 (Plasma Kallikrein-sensitive glycoprotein) variant, AGT, DBP, KNG1, SERPINA 3, LRG1, C4B, HP, CLU, PON3, C3, CD5L, SHBG, FGG, APOL1, CFHR1, FGB, TF, ALB, CFI, IGHM, ALB isoform CRA_k, IGKVA-1, MBL2, TTR, IGKC, APOM, SERPINA 1, CLEC3B	[78]
Acute heart failure (HF)	Exosome isolation kit	Serum	Human, n = 43	Electron microscopy Nanoparticle tracking analysis Western Blotting	CD63 and Hsp70 expression	miR-92b-5p, miR-192-5p, miR-320a	[79]
	Exosome isolation kit	Serum	Human, n = 28	Electron microscopy Nanoparticle tracking analysis Western blotting	Size: 40–150 nm (average 80 nm); CD63 and Hsp70 expression	miR-92b-5p, miR-192-5p, miR-320a	[80]
HF	ExoQuick Exosome Precipitation Solution	Serum	Human, n = 4	Western Blotting	CD63 expression	miR-192, miR-194, miR-34a	[81]
	Ultracentrifugation	Pericardial fluid	Human, n = 51	n. d.	n. d.	miR-210, let-7b-3p, let-7d-3p, miR-1, miR-125a-5p, miR-126-3p, miR-129-5p, miR-132-3p, miR-133a, miR-135a-5p, miR-135b-5p, miR-138-5p, miR-139-5p, miR-140-5p, miR-143-3p, miR-145-5p, miR-146a-3p, miR-146a-5p, miR-17-5p, miR-181a-5p, miR-181b-5p, miR-181c-5p, miR-208a, miR-20a-5p, miR-21-3p, miR-214-3p, miR-23a-3p, miR-23b-3p, miR-25-3p, miR-30a-3p, miR-30c-5p, miR-30e-3p, miR-320a, miR-330-5p, miR-339-3p, miR-346, miR-34c-3p, miR-365a-3p, miR-375, miR-499a-5p, miR-505-3p, miR-532-3p, miR-671-5p, miR-92b-3p, miR-9-3p	[82]
	Exosome Isolation kit	Plasma	Human, n = 40	n. d.	n. d.	miR-486, miR-146a	[83]
Idiopathic pulmonary arterial hypertension (IPAH)	Ultracentrifugation	Plasma	Human, n = 5	Nanoparticle tracking analysis BCA Protein assay Immunoblotting	CD31, CD63 and TSG101 expression	n. d.	[84]
	Ultracentrifugation	Plasma	Human, n = n. d.	Nanoparticle tracking analysis Transmission electron microscopy Western blotting	n. d.	miR-let-7c, miR-let-7d, miR-16, miR-18a, miR-19b, miR-20a, miR-20b, miR-27b, miR-30b, miR-30c, miR-125a-5p, miR-145, miR-146b. miR-148a, miR-195, miR-200b, miR-215, miR-218, miR-221, miR-339-3p, miR-365	[85]
Arterial disease/ cardiovascular risk factors	ExoQuick Exosome Precipitation Solution	Plasma	Human, n = 1012	BCA Protein assay	n. d.	n. d.	[86]
(i) Hypertension	Exosome isolation kit	Plasma	Spontaneous hypertensive rats (SHRs), n = n. d.	Dynamic light scattering Western blotting	Size: 10–200 nm diameter (those ranging 30–150 nm accounted for 80%); CD63 and Hsp70 expression	rno-miR-148a-3p, rno-miR-122-5p, rno-miR-143-3p, rno-miR-192-5p, rno-let-7i-5p, rno-miR-215, rno-miR-140-3p, rno-miR-99a-5p, rno-miR-6329, rno-miR-378a-3p, rno-miR-486, rno-miR-378a-5p, rno-miR-6328, rno-miR-187-3p, rno-miR-383-5p, rno-miR-206-3p, rno-miR-425-5p, rno-miR-128-3p, rno-miR-181c-3p, rno-let-7d-5p, rno-miR-191a-3p, rno-miR-185-5p, rno-miR-218a-5p, rno-let-7f-5p, rno-miR-148a-5p, rno-miR-322-3p, rno-miR-181d-5p, rno-miR-223-5p, rno-miR-191a-5p, rno-miR-17-5p, rno-miR-3559-5p, rno-let-7a-5p, rno-miR-15b-5p, rno-miR-223-3p, rno-miR-872-5p, rno-miR-3068-3p	[87]
	Ultracentrifugation	Serum	Cardiac hypertrophic Wistar rats, n = 6	Electron microscopy Western blotting	n. d.	HSP90, HSC70, CD63, CD9, GAPDH, CD68, miR-17-3p, miR-145-5p, miR-221-3p, miR-222-5p	[88]
	n. d.	Urine	C57BL6J/Ola mice, n = n. d.	Nanoparticle tracking analysis Western blotting	n. d.	NCC	[89]
	Ultracentrifugation	Urine	Human, n = 11	n. d.	n. d.	RAIG-2, SDCBP, NKCC2, TSC, ACTB, RAIG-3, ANPEP, GAPDH, MME, EZR, KRT1, ENO1, LDHB, HSPA8, ANXA2	[90]
(ii) Obesity	n. d.	Plasma	Human, n = 23	n. d.	n. d.	miR-122	[91]
(iii) Type 2 diabetes (T2D)	ExoQuick Exosome Precipitation Solution	Serum	Human, n = 33	n. d.	n. d.	miR-122-5p, let-7a-3p, miR-26b-3p, miR-193b-5p, miR-4532, miR-432-5p, let-7f-1-3p, miR-183-5p, miR-3656, miR-340-3p, miR-6751-3p, miR-1273a, miR-4484, miR-8485, miR-4644, miR-1273g-3p, miR-4271, miR-7847-3p, miR-4461, miR-6885-5p	[92]
	ExoQuick Exosome Precipitation Solution	Plasma	Human, n = 18	n. d.	n. d.	miR-326, miR-532-5p, miR-186, miR-127-3p, let-7g, let-7d, miR-126, miR-101, miR-18b, miR-21, miR-199a-3p, miR-502-3p, miR-495, miR-132, miR-15b, miR-200c, miR-223-5p, miR-16, miR-543, miR-195, let-7a, miR-26b, miR-374a, miR-26a, let-7f, ADIPOR1, ADIPOR2, APPL1	[93]
(iv) Diabetic nephropathy	ExoQuick Exosome Precipitation Solution	Serum	Human, n = 33	n. d.	n. d.	miR-122-5p, miR-432-5p, miR-3656, miR-193b-5p, miR-6087, miR-4488, miR-26b-3p, miR-8485, miR-23a-5p, miR-4532, let-7a-3p, miR-6739-5p, miR-1273a, miR-7641, miR-4461, miR-6751-3p, miR-4484, miR-7847-3p, miR-1273g-3p, miR-140-5p	[92]
(v) Familial hypercholesterolemia (with a CV event)	Filtration	Plasma	Human, n = 42	Nanoparticle tracking analysis Flow cytometry (CD63 and CD81)	n. d.	miR-130b, miR-133a, miR-142-3p, miR-200c, miR-324-5p, miR-339-3p, miR-425-5p, miR-660, miR-744, miR-122	[94]
(vi) Obstructive sleep apnea (OSA)	Exosome isolation kit	Plasma	Human, n = 8	Electron microscopy Western blotting	n. d.	hsa-miR-16-5p, hsa-miR-4459, hsa-miR-451a, hsa-miR-6510-5p	[95]
	Exosome isolation kit	Plasma	Human, n = n. d.	Electron microscopy Flow cytometry Western blotting	CD63 expression	n. d.	[96]
	Exosome isolation kit	Plasma	Human, n = 10	Electron microscopy	n. d.	n. d.	[97]
Myocardial ischemia/reperfusion (IR)	ExoQuick Exosome Precipitation Solution	Plasma	Human, n = 4	Transmission electron microscopy BCA Protein assay Flow cytometry Western blotting	Cup-shaped membrane-bound vesicles;size: ~100 nm diameter; CD63, CD9 and CD81 expression	miR-24, miR-21, miR-214, miR-132, miR-195, miR-210, miR-144, miR-150, miR-34a	[98]
	Ultracentrifugation	Serum	MI Wistar rats, n = 3	BCA Protein assay Western blotting	CD9 and Hsp90 expression	miR-21, miR-29a, miR-30a, miR-133a	[99]
Coronary artery bypass graft (CABG)	Column-based system	Plasma	Human, n = 21	Nanoparticle tracking analysis Transmission electron microscopy Western blotting	n. d.	miR-1, miR-23a, miR-24, miR-92a, miR-126, miR-133a, miR-133b, miR-208a, miR-208b, miR-210, miR-223, miR-451	[100]

Legend: CAD, coronary artery disease; MYBPC3, myosin-binding protein C, cardiac-type; VIM, vimentin; LTQ, linear trap quadrupole; AMI, acute myocardial infarction; Rab 5B, Ras-related protein Rab-5B; Apo-J, apolipoprotein J; KD, Kawasaki disease; ITIH4, inter-alpha-trypsin inhibitor heavy chain H4; PROS1, vitamin K-dependent protein S; C9, complement component C9; AFM, afamin; A1BG, alpha-1B-glycoprotein; IGFALS, insulin-like growth factor-binding protein complex acid labile subunit; C4A, complement C4-A; HPX, hemopexin; SERPINC 1, antithrombin-III; AGT, angiotensinogen; DBP, vitamin D-binding protein; KNG1, kininogen-1; SERPINA 3, alpha-1 antichymotrypsin; LRG1, leucine-rich alpha-2-glycoprotein; C4B, complement C4-B; HP, haptoglobin; CLU, clusterin; PON3, serum paraoxonase/lactonase 3; C3, complement C3; CD5L, CD5 antigen-like; SHBG, sex hormone-binding globulin; FGG, fibrinogen gamma chain; APOL1, apolipoprotein L1; CFHR1, complement factor H-related protein 1; FGB, fibrinogen beta chain; TF, serotransferrin; ALB, serum albumin; CFI, complement factor I; IGHM, immunoglobulin heavy constant mu; IGKVA-1, immunoglobulin kappa variable 4-1; MBL2, mannose-binding protein C; TTR, transthyretin; IGKC, immunoglobulin kappa constant; APOM, apoliprotein M; SERPINA 1, alpha-1-antitrypsin; CLEC3B, tetranectin; HF, heart failure; DCM, dilated cardiomyopathy; HSP70, heat shock protein 70; IPAH, idiopathic pulmonary arterial hypertension; BCA, bicinchoninic acid; TSG101, tumor susceptibility gene 101 protein; SHRs, spontaneous hypertensive rats; HSP90, heat shock protein 90; GAPDH, glyceraldehyde 3-phosphate dehydrogenase; NCC, sodium chloride cotransporter; RAIG-2, retinoic acid-induced gene 2 protein; SDCBP, syntenin-1; NKCC2, solute carrier family 12 member 1; TSC, solute carrier family 12 member 3; ACTB, actin, cytoplasmic 1; RAIG-3, G-protein coupled receptor family C, group 5, member C; ANPEP, aminopeptidase N; MME, neprilysin; EZR, ezrin; KRT1, keratin, type II cytoskeletal 1; ENO1, alpha-enolase; LDHB, L-lactate dehydrogenase B chain; HSPA8, Heat shock cognate 71 kDa protein; ANXA2, Annexin A2; T2D, type 2 diabetes; ADIPOR1, adiponectin receptor 1; ADIPOR2, adiponectin receptor 2; APPL1, DCC-interacting protein 13-alpha; CV, cardiovascular; OSA, obstructive sleep apnea; IR, ischemia/reperfusion; CABG, coronary artery bypass graft; n. d., not described.

**Table 3:** miRNAs found to be upregulated in coronary artery disease and heart failure patients.

miRNA	Cardiovascular Disease	Variation	Included/Not Included in the Vesiclepedia
miR-133a	Coronary artery disease (CAD)	+	Included
miR-208a	Coronary artery disease (CAD)	+	Not included
miR-1	Coronary artery disease (CAD)	+	Not included
miR-499-5p	Coronary artery disease (CAD)	+	Not included
miR-92b-5p	Coronary artery disease (CAD)	+	Not included
miR-30a	Coronary artery disease (CAD)	+	Not included
miR-192	Heart failure (HF)	+	Included
miR-194	Heart failure (HF)	+	Included
miR-146a	Heart failure (HF)	+	Included
miR-92b-5p	Heart failure (HF)	+	Not included

Legend: CAD, coronary artery disease; HF, heart failure; +, upregulated.

**Table 4:** Proteins found to be up- and downregulated in coronary artery disease patients.

Gene Name	Cardiovascular Disease	Variation	Included/Not Included in the Vesiclepedia
AGT	Coronary artery disease (CAD)	+	Included
C4B	Coronary artery disease (CAD)	+	Included
HP	Coronary artery disease (CAD)	+	Included
FGG	Coronary artery disease (CAD)	+	Included
FGB	Coronary artery disease (CAD)	+	Included
VIM	Coronary artery disease (CAD)	+	Not included
ITIH4	Coronary artery disease (CAD)	+	Not included
C9	Coronary artery disease (CAD)	+	Not included
IGKC	Coronary artery disease (CAD)	+	Not included
MYBPC3	Coronary artery disease (CAD)	+	Not included
SERPINA3	Coronary artery disease (CAD)	+	Not included
LRG1	Coronary artery disease (CAD)	+	Not included
CFHR1	Coronary artery disease (CAD)	+	Not included
IGKVA-1	Coronary artery disease (CAD)	+	Not included
PROS1	Coronary artery disease (CAD)	−	Included
C4A	Coronary artery disease (CAD)	−	Included
A1BG	Coronary artery disease (CAD)	−	Included
KNG1	Coronary artery disease (CAD)	−	Included
CLU	Coronary artery disease (CAD)	−	Included
C3	Coronary artery disease (CAD)	−	Included
CD5L	Coronary artery disease (CAD)	−	Included
APOL1	Coronary artery disease (CAD)	−	Included
TF	Coronary artery disease (CAD)	−	Included
ALB	Coronary artery disease (CAD)	−	Included
MBL2	Coronary artery disease (CAD)	−	Included
TTR	Coronary artery disease (CAD)	−	Included
SERPINA1	Coronary artery disease (CAD)	−	Included
AFM	Coronary artery disease (CAD)	−	Not included
IGFALS	Coronary artery disease (CAD)	−	Not included
HPX	Coronary artery disease (CAD)	−	Not included
SERPINC1	Coronary artery disease (CAD)	−	Not included
DBP	Coronary artery disease (CAD)	−	Not included
CFI	Coronary artery disease (CAD)	−	Not included
IGHM	Coronary artery disease (CAD)	−	Not included
APOM	Coronary artery disease (CAD)	−	Not included
CLEC3B	Coronary artery disease (CAD)	−	Not included
PON3	Coronary artery disease (CAD)	−	Not included
SHBG	Coronary artery disease (CAD)	−	Not included

Legend: CAD, coronary artery disease; AGT, angiotensinogen; C4B, complement C4-B; HP, haptoglobin; FGG, fibrinogen gamma chain; FGB, fibrinogen beta chain; VIM, vimentin; ITIH4, inter-alpha-trypsin inhibitor heavy chain H4; C9, complement component C9; IGKC, immunoglobulin kappa constant; MYBPC3, myosin-binding protein C, cardiac-type; SERPINA3, alpha-1 antichymotrypsin; LRG1, leucine-rich alpha-2-glycoprotein; CFHR1, complement factor H-related protein 1; IGKVA-1, immunoglobulin kappa variable 4-1; PROS1, vitamin K-dependent protein S; C4A, complement C4-A; A1BG, alpha-1B-glycoprotein; KNG1, kininogen-1; CLU, clusterin; C3, complement C3; CD5L, CD5 antigen-like; APOL1, apolipoprotein L1; TF, serotransferrin; ALB, serum albumin; MBL2, mannose-binding protein C; TTR, transthyretin; SERPINA1, alpha-1-antitrypsin; AFM, afamin; IGFALS, insulin-like growth factor-binding protein complex acid labile subunit; HPX, hemopexin; SERPINC1, antithrombin-III; DBP, vitamin D-binding protein; CFI, complement factor I; IGHM, immunoglobulin heavy constant mu; APOM, apoliprotein M; CLEC3B, tetranectin; PON3, serum paraoxonase/lactonase 3; SHBG, sex hormone-binding globulin; +, up-regulated; −, down-regulated.

## Data Availability

The data that supports the findings of the study are available in the Supplementary Material of this article.

## Supplementary Materials

The following are available online at https://www.mdpi.com/2227-7382/9/1/8/s1, Table S1: Selection of the studies according to the three levels of exclusion criteria.; Table S2: Description of the studies selected for analysis. Exosome and exosome-based mediators and respective variation, values and isolation method.; Table S3: Top 10 target genes of exosome-based miRNAs, when available, using TarBase v.8 software from DIANA tools (http://carolina.imis.athena-innovation.gr/diana_tools/web/index.php?r=tarbasev8%2Findex). Acessed on 18/01/2021.; Table S4: Exosome-based miRNAs variation in plasma and serum samples of coronary artery disease patients. These miRNAs were enrolled in the FunRich analysis to evaluate if they are included or not included in the Vesiclepedia.; Table S5: Vesiclepedia database on exosome-derived miRNAs from serum and plasma human samples (accessed on 6th July 2020).; Table S6: Exosome-based protein variation in plasma and serum samples of coronary artery disease patients. These proteins were enrolled in the FunRich analysis to evaluate if they are included or not included in the Vesiclepedia.; Table S7: Vesiclepedia database on exosome-derived proteins from serum and plasma human samples (accessed on 7 July 2020).
